# Improved method for optical coherence tomography angiography: from reconstruction to clinical indicator quantification

**DOI:** 10.1117/1.JBO.31.5.056002

**Published:** 2026-05-17

**Authors:** Fuxin Cai, Xianglong Feng, Zhihong Zheng, Yuhong Zhang, Duo Xu, Chuanwei Ma, Yu Fan, Xinjian Chen

**Affiliations:** a Suzhou Big Vision Medical Imaging Technology Co., Ltd., Suzhou, China; b Southern Medical University, School of Biomedical Engineering, Guangzhou, China; c Soochow University, School of Electronics and Information Engineering, Suzhou, China; d University of Electronic Science and Technology of China, School of Medicine, Chengdu, China; e Sichuan Provincial People’s Hospital, Eye Institute, Chengdu, China

**Keywords:** optical coherence tomography angiography, split spectrum, Walsh function, speckle contrast, local fractal dimension, clinical quantitative index

## Abstract

**Significance:**

Optical coherence tomography angiography (OCTA) represents a significant advance in noninvasive ophthalmic vascular imaging, yet existing reconstruction algorithms face challenges such as spectral leakage and motion artifacts, which can compromise image quality and the accuracy of subsequent clinical quantification. Improving OCTA reconstruction and developing robust, automated methods for clinical indicator extraction are crucial for enhancing diagnostic reliability and facilitating precise disease monitoring.

**Aim:**

We aim to propose and validate an improved OCTA reconstruction method based on a smoothed Walsh window function to reduce spectral leakage while preserving axial resolution, coupled with an enhanced blood flow B-scan signal using retinal layer segmentation. Furthermore, we seek to develop and evaluate a fully automated pipeline for calculating key clinical indicators—including foveal avascular zone (FAZ) parameters and vessel density—based on local fractal dimension analysis.

**Approach:**

A spectral-domain OCT system was used to acquire volumetric retinal data from healthy volunteers. The reconstruction method utilized a smoothed Walsh window for full-spectrum splitting to mitigate spectral leakage, combined with a deep learning-based retinal layer segmentation algorithm and Otsu’s thresholding to enhance blood flow B-scan signals. For clinical quantification, local fractal dimension analysis was employed to segment vascular networks and FAZ regions automatically, from which perimeter, area, circularity index, and sectoral vessel density were computed.

**Results:**

The proposed reconstruction method demonstrated superior performance compared with traditional OMAG and SSADA algorithms, showing significant improvements in vessel connectivity, contrast (increase up to ∼0.74), and signal-to-noise ratio (SNR increase up to ∼1.09  dB). The FAZ segmentation based on local fractal dimension achieved a high similarity index of 0.9735±0.0066 with manual ground truth, with low false positive (1.76%±0.75%) and false negative (3.48%±0.78%) rates. Intraclass correlation coefficients for FAZ area, perimeter, and circularity index all exceeded 0.90. Vessel density measurements across retinal sectors were consistent with established normative data.

**Conclusions:**

The integration of a smoothed Walsh window function and retinal layer segmentation significantly enhances OCTA image quality and blood flow signal clarity. The local fractal dimension-based automated analysis pipeline provides accurate, reproducible quantification of FAZ morphology and vessel density, demonstrating strong agreement with manual annotations. This method offers a reliable framework for improving both OCTA reconstruction and the automated derivation of clinical indicators, supporting advanced ophthalmic diagnosis and longitudinal disease assessment.

## 1 Introduction

Optical coherence tomography angiography (OCTA) is a rapidly developing noninvasive vascular imaging technology that represents a significant breakthrough in the field of ophthalmic diagnosis. As a functional extension based on optical coherence tomography (OCT), OCTA can achieve high-resolution three-dimensional visualization of the microvascular network by detecting blood flow signals without the need for contrast agent injection. It has advantages such as high resolution, high sensitivity, and noninvasiveness.^1^^–^^4^ Since its emergence, this technology has been rapidly applied in the diagnosis and follow up of various ophthalmic diseases, promoting the transformation of ophthalmic imaging diagnostic models.

In the field of biomedical imaging, vascular imaging has long relied on traditional methods that utilize contrast agents for enhancement, such as fundus fluorescein angiography (FFA) and indocyanine green angiography (ICGA).^5^^–^^8^ Although these techniques are regarded as the gold standard, they have significant limitations that cannot be ignored. Approximately 1% to 5% of patients may experience adverse reactions such as nausea, vomiting, allergic reactions, and even anaphylactic shock after receiving the contrast agent, and the examination process takes a considerable amount of time, requiring 5 to 10 min of preparation.^9^ Moreover, traditional vascular imaging techniques are unable to obtain deep-layer information and cannot accurately distinguish vascular lesions at different layers of the retina and choroid.

The technical principle of OCTA is based on OCT. OCT is a noninvasive cross-sectional imaging technique that utilizes the principle of weakly coherent light interference. By detecting the signals of backscattering or scattering of light in different depth layers of biological tissues, it can obtain two-dimensional or three-dimensional structural images of biological tissues,^10^^–^^13^ with a resolution of micrometer level. OCTA, on this basis, repeatedly scans the same position multiple times to detect the signal changes caused by the movement of red blood cells and extracts vascular information based on the motion contrast mechanism. Compared with traditional OCT, OCTA not only retains the ability to image tissue structure but also adds functional information such as hemodynamic and microvascular morphology.^14^^–^^17^

The OCTA algorithm has undergone an evolution process from simple to complex. The early algorithms mainly relied on the changes in amplitude (intensity) signals, such as split-spectrum amplitude decorrelation angiography (SSADA), optical microangiography based on intensity signals (OMAG-A), and speckle contrast angiography (ISCA).^18^^–^^21^ They detected blood flow by calculating the correlation values or differences of the amplitude signals between consecutive B-scan images. These algorithms were sensitive to amplitude signal changes but had limited resistance to motion artifacts such as eye movements. With technological advancements, phase information was introduced into the OCTA algorithm. Phase signals are more sensitive to small movements and can detect lower-speed blood flow, such as phase gradient angiography (PGA) and phase variance angiography (Phase-PVA).^22^^,^^23^ However, phase algorithms are also more sensitive to sample motion and system noise, which may lead to more artifacts. To overcome the limitations of a single signal, modern OCTA systems often adopt composite algorithms, using both amplitude and phase information. They improve the sensitivity and specificity of blood flow detection through methods such as complex difference and cross-correlation, such as optical microangiography based on complex signals (OMAG-C), compound variance angiography (CDV), and inverse signal-to-noise ratio decorrelation angiography (ISNR-ID).^4^^,^^24^^–^^26^

Despite their enhanced sensitivity to slow blood flow, algorithms incorporating phase information exhibit critical limitations.^22^^–^^25^ Phase signals are hyper-sensitive to bulk tissue motion, such as involuntary eye movements, which introduce severe motion artifacts and necessitate computationally expensive phase compensation procedures. Furthermore, phase instability in noisy or defocused regions can significantly degrade angiographic contrast. Consequently, amplitude-based decorrelation methods, particularly SSADA, remain highly valuable in clinical settings due to their inherent robustness against motion artifacts and superior computational efficiency ^18^^,^^22^. However, the traditional SSADA algorithm faces its own structural bottleneck as it utilizes Gaussian windows to partition the optical spectrum. As Gao et al.^18^ noted, this spectrum-splitting approach inherently narrows the effective bandwidth of each spectral split, leading to a substantial degradation in axial resolution. Overcoming these resolution and leakage issues remains a critical challenge in the development of high-fidelity amplitude-based OCTA.

The quantitative analysis capabilities of OCTA provide objective indicators for disease assessment. Using specialized software, parameters such as vessel density, nonperfusion area, macular foveal nonvascular area, and morphology of OCTA images can be automatically calculated.^1^^,^^27^ These quantitative indicators reduce the errors in subjective judgment and facilitate the precise monitoring of disease progression and treatment responses. Studies have shown that the vessel density of patients with diabetic retinopathy is negatively correlated with the severity of the disease.^1^

In light of the aforementioned issues, this study proposes an improved OCTA reconstruction method coupled with a fully automated pipeline for clinical indicator quantification. The specific originality and contributions of this work are threefold: First, to address the axial resolution degradation caused by traditional Gaussian windows, we propose a novel spectrum-splitting method utilizing a smoothed Walsh window function. This approach utilizes the full spectrum to effectively mitigate spectral leakage caused by abrupt signal transitions, without significantly compromising the axial resolution of the system. Second, rather than claiming the segmentation algorithm itself as a novelty, we propose an innovative signal enhancement strategy that integrates an established deep-learning-based retinal layer segmentation algorithm with a statistical-based thresholding method. By repurposing the segmented layers as a dynamic anatomical mask to identify nonretinal regions, this strategy strictly truncates background noise signals and significantly enhances the intensity of the OCT blood flow B-scans prior to projection. Finally, based on the enhanced reconstructions, we develop a fully automated pipeline for clinical indicator extraction. We employ local fractal dimension (LFD) analysis to automatically segment vascular networks and FAZ regions, from which key clinical parameters—including perimeter, area, circularity index, and sectoral vessel density—are objectively computed. To validate this integrated approach, 3D volumetric data of healthy human retinas were acquired using a custom-built OCT system. Experimental results demonstrate that our method significantly improves OCTA image quality, outperforming traditional OMAG and SSADA in terms of signal-to-noise ratio (SNR), contrast, and vessel connectivity.

## 2 OCTA Reconstruction Method based on Improved SSADA and Its Clinical Indicators

### 2.1 Reconstruction Principle of OCTA

OCT detects the interference signal between the reflected signals from the reference mirror and the biological sample, and then, through a series of reconstruction steps such as interpolation and Fourier transformation, it finally generates the structural diagram of the sample. The OCT signal is essentially a complex function containing amplitude and phase information, which can be expressed by the following equation:^1^

$$\tilde{E}(x,z)=A(x,z)\cdot \exp(i\cdot \varphi (x,z)),$$ (1)

where A(x,z) represents the amplitude of the OCT signal in the lateral x direction and depth z, and φ(x,z) represents the phase of the OCT signal in the lateral x direction and depth z.

#### 2.1.1 Principle of the SSADA algorithm

The SSADA algorithm processes the collected raw data along the wave number direction using multiple Gaussian windows for different bands of raw data. Then, it calculates the decorrelation signals of the data collected at the same band and at different times at the same location, obtaining multiple decorrelation signals of different bands at the same location. After averaging these signals, a thresholding method is adopted to extract the final blood flow signal. The specific calculation method of the decorrelation signals $D_{\mathrm{SSADA}}(x,z)$ based on Eq. (1) is as follows:

$$D_{\mathrm{SSADA}}(x,z)=\frac{1}{N-1}\cdot \frac{1}{M}\overset{N-1}{\underset{n=1}{\sum }}\overset{M}{\underset{m=1}{\sum }}\{1-\frac{A_{m,n}(x,z)A_{m,n+1}(x,z)}{\left[\frac{1}{2}A_{m,n}(x,z{)}^{2}+\frac{1}{2}A_{m,n+1}(x,z{)}^{2}\right]}\},$$ (2)

where M represents the number of spectral bands and N represents the repetition times of B-scan at the same position. The value of $D_{\mathrm{SSADA}}(x,z)$ ranges from 0 to 1, and its magnitude indicates the degree of correlation between different B-scan collected at the same position at different times; the smaller the $D_{\mathrm{SSADA}}(x,z)$, the lower the correlation between the B-scan; conversely, the higher the correlation.^18^

#### 2.1.2 Principle of ISCA

The ISCA obtains an improved speckle contrast image by calculating the ratio of the standard deviation between B-scan data collected at different times at the same location to the average intensity. To eliminate the high intensity values of static tissues and enhance the blood flow signal, it uses multiple averaged B-scan images and the speckle contrast image for masking processing to generate the final blood flow image. The specific calculation method for the improved ISC blood flow signal $I_{\text{flow}}(x,z)$ based on Eq. (1) is as follows:

$$I_{\text{flow}}(x,z)=\frac{1}{N-1}\cdot \frac{1}{N}\cdot \overset{N-1}{\underset{n=1}{\sum }}\frac{\left|A_{n+1}(x,z)-A_{n}(x,z)\right|}{A_{n+1}(x,z)\cdot A_{n}(x,z)}\cdot \overset{N}{\underset{n=1}{\sum }}A_{n}(x,z),$$ (3)

where N still represents the number of repetitions of the B-scan at the same location. The value of $I_{\text{flow}}(x,z)$ indicates the intensity of the blood flow signal at that location; the smaller the $I_{\text{flow}}(x,z)$, the lower the intensity of the blood flow signal, and conversely, the higher the intensity.^21^

#### 2.1.3 Construction of the window function for split-spectrum

The SSADA algorithm employs Gaussian windows to partition the full OCT spectrum into multiple narrow bands, yielding images with a reduced axial resolution that is comparable to the lateral resolution. Although spectral splitting methods based on Gaussian, Hanning, and Kaiser windows can accurately capture variations in blood flow signals, they inherently discard a portion of the data along the A-scan direction, leading to a degradation in the axial resolution of the reconstructed images. Consequently, this study utilized an improved Walsh function to generate window functions that cover the entire spectrum, thereby ensuring the accurate extraction of blood flow signals while minimizing the loss of axial resolution.^28^

**Fig. 1:**
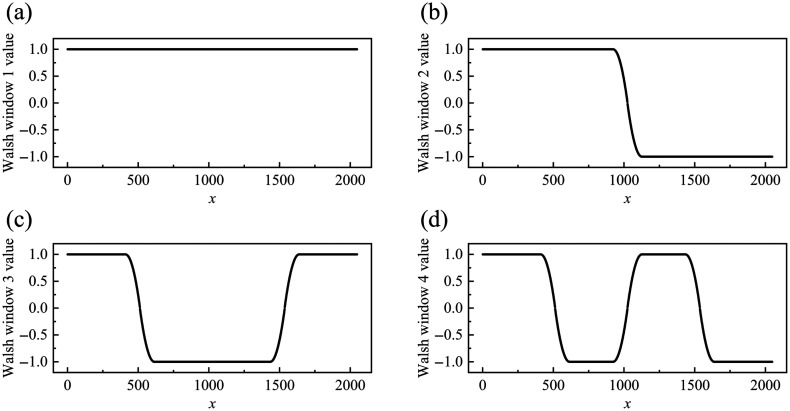
Function graph of the first four Walsh windows. (a) The first Walsh window function. (b) The second Walsh window function. (c) The third Walsh window function. (d) The fourth Walsh window function.

As the values of the Walsh function are only 1 and −1, when constructing the window function using it and applying the window to the collected original spectral data, it will cause positive and negative jumps at certain points in the original spectral signal. This results in an increase in spectral leakage after the fast Fourier transform (FFT), thereby affecting the extraction of blood flow. Therefore, in this paper, an arctangent function was constructed at the 5% points on both sides of the discontinuity points of the Walsh function’s jumps to smooth the Walsh window function, thereby reducing the impact of spectral leakage on the final blood flow extraction. The formula of the Walsh window function Walsh(n,x) constructed in this paper is as follows:^29^

$$\text{Walsh}(n,x)=\{\begin{array}{cc} \overset{3}{\underset{i=0}{\prod }}\{\text{sign}[\sin(2^{i+1}\pi x)]{\}}^{g_{i}}, & x\notin (p_{m}-\alpha \cdot N,p_{m}+\alpha \cdot N)\\ \pm \frac{2}{\pi }\;\arctan[\beta \cdot (\frac{x-p_{m}}{\alpha \cdot N})], & x\in (p_{m}-\alpha \cdot N,p_{m}+\alpha \cdot N) \end{array},$$ (4)

where n represents a decimal number; $g_{i}$ represents the i’th bit of the Gray code corresponding to the decimal number n; $p_{m}$ represents the position of the m’th jump discontinuity point of the Walsh function; and α is the truncation factor on the left and right of the jump discontinuity point of the Walsh function, which controls the width of the transition region. In this paper, its value was empirically set to 0.01 for our specific system setup. β is the scaling coefficient of the arctangent function along the x-axis, which controls the steepness of the smoothing curve, and its value was taken as 2. It is important to note that the selection of these aforementioned coefficients is not rigidly fixed but is systematically determined based on the mathematical properties of the arctangent function combined with the spectral sample size (N) of the current OCT system. The primary optimization criterion for determining α and β is to ensure that over 90% of the amplitude variation is completed within the defined transition region. Consequently, when applying this method to other OCT systems with different spectrometer resolutions, α and β should be adaptively adjusted to satisfy this transition criterion. N represents the length of the data; sign[⋅] is the sign function, and its specific expression can be

$$\text{sign}[f(x)]=\{\begin{array}{cc} +1, & f(x)>0\\ -1, & f(x)<0 \end{array},$$ (5)

where f(x) is the variable of the sign function. In this paper, when constructing the window function, as the values of the first Walsh function are all 1, in the actual blood flow extraction algorithm, only the first 2, 3, and 4 Walsh functions were taken for calculation. The window function image improved based on the first four Walsh functions constructed in this paper is shown in Fig. 1. Figures 1(a)–1(d) are the window function images constructed based on the first four Walsh functions, respectively.

#### 2.1.4 Algorithmic process of angiography reconstruction

**Fig. 2:**
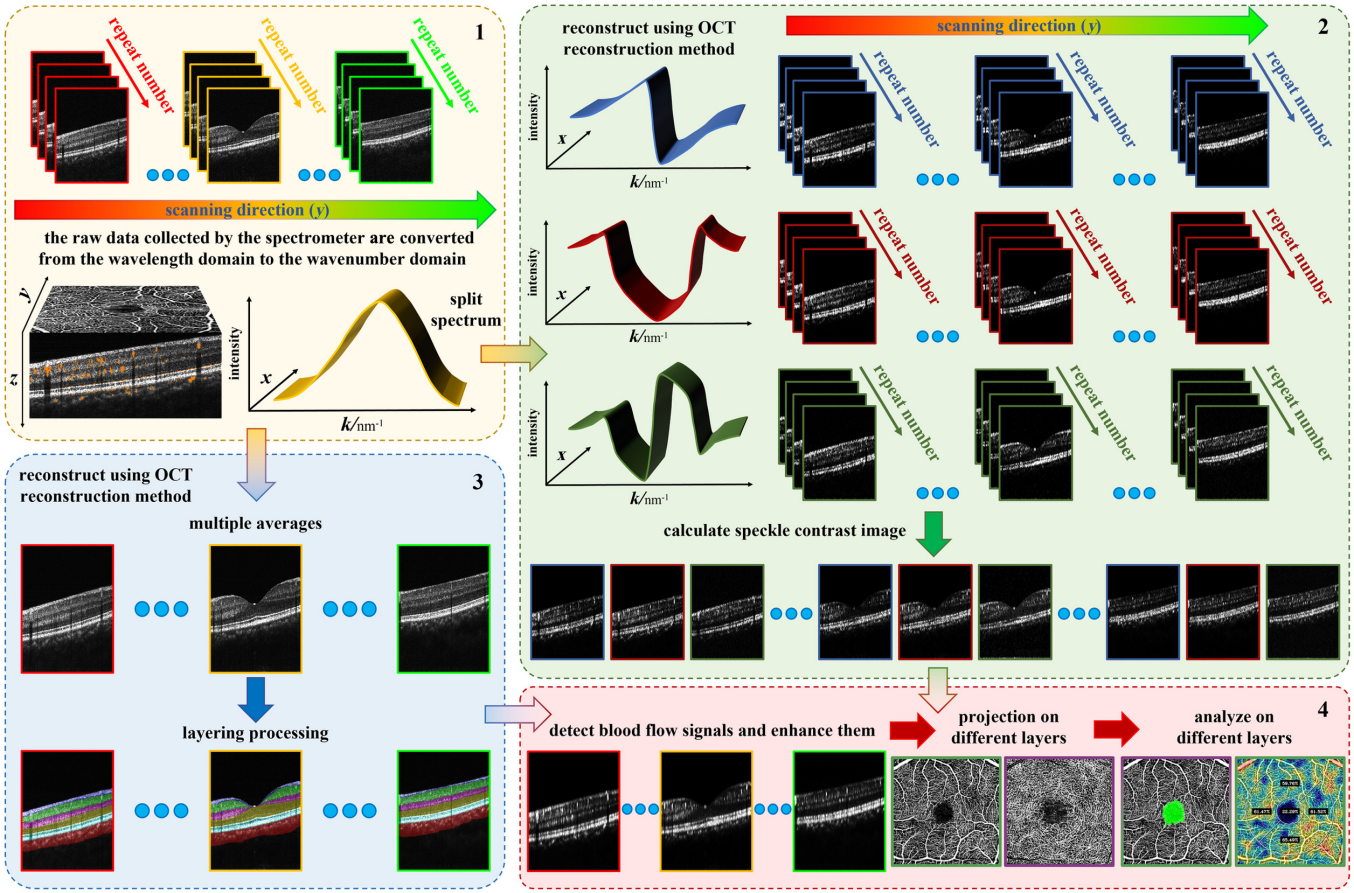
Flow chart of the complete OCTA processing pipeline. Block 1 illustrates the standard data acquisition protocol (a prerequisite step), whereas blocks 2, 3, and 4 represent the three specific stages of the proposed angiography reconstruction algorithm.

The complete OCTA processing pipeline is illustrated in Fig. 2. Prior to algorithmic reconstruction, standard volumetric data acquisition was performed, involving four repeated B-scans at each lateral position. The subsequent reconstruction procedure is characterized by three synergistic processing stages.

Walsh-window-based spectral division was first executed along the wavenumber direction. The raw spectral data were partitioned using the smoothed second, third, and fourth Walsh window functions, resulting in three distinct spectral subsets. These subsets were processed into depth-resolved amplitude signals using a standard SD-OCT reconstruction pipeline—encompassing background subtraction, interpolation, and fast Fourier transform.^22^ To mitigate motion-induced instability, the phase compensation method from Ref. 24 was directly applied. Following reconstruction, speckle contrast was calculated for each subset according to Eq. (3) to generate three dynamic signal volumes.

**Fig. 3:**
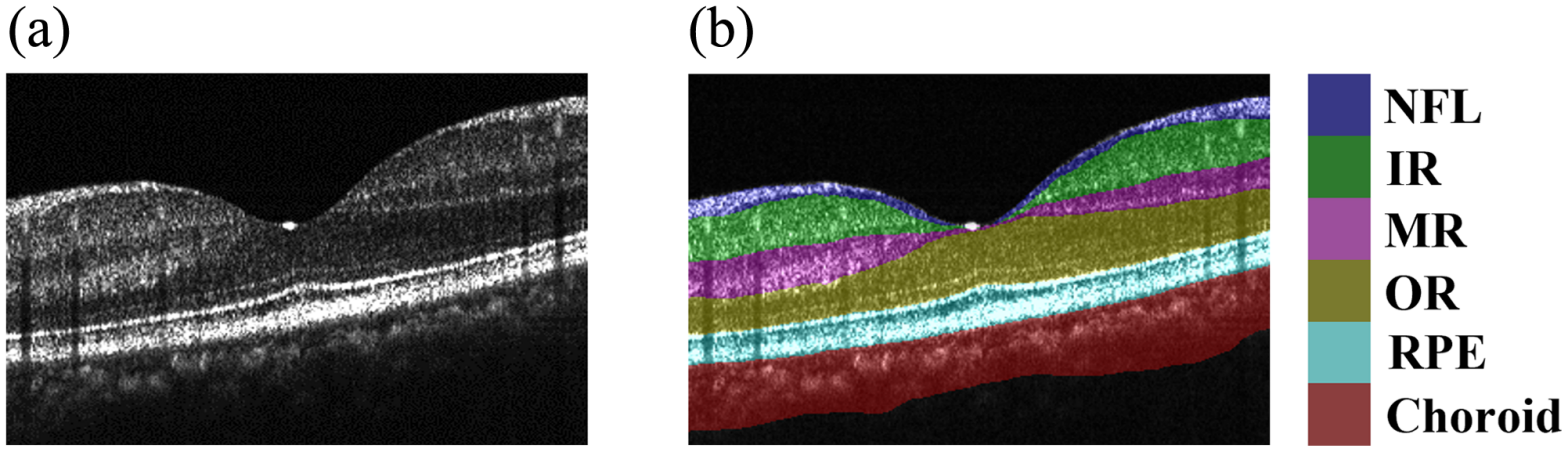
OCT layering diagram. (a) OCT image without layering. (b) Mapping of layered results on the OCT image.

Structural reconstruction and layer segmentation were performed simultaneously using the full-spectrum raw data.^18^ To improve the signal-to-noise ratio and structural contrast, multiple frames at the same location were averaged to produce a high-quality structural volume.^18^ As illustrated in the OCT layering scheme in Fig. 3, the deep learning-based MIAS-3000 algorithm was then utilized to delineate the retina into distinct anatomical layers (from NFL to Choroid). Specifically, Fig. 3(a) presents an unsegmented OCT cross-section, whereas Fig. 3(b) displays the resulting segmentation boundaries mapped onto the structural image. This step provided the necessary anatomical constraints for subsequent signal refinement. Although the deep-learning-based MIAS-3000 algorithm was employed in this study for its high precision, it is important to note that the proposed signal enhancement strategy is algorithm-agnostic. The anatomical mask can be derived from any high-quality OCT layer segmentation, ensuring the versatility of our reconstruction framework across different clinical platforms.

Mask-assisted flow enhancement and en face projection constituted the final stage. The dynamic signal volumes and the structural segmentation results were integrated such that the segmented retinal boundaries provided a dynamic spatial template to isolate valid flow regions. Otsu’s thresholding^30^ was then applied to these isolated regions to truncate residual noise and enhance the blood flow B-scan intensity. Finally, en face angiograms were generated via mean projection along the depth (z) direction. As background noise was strictly suppressed by the layer-masking and thresholding procedures prior to projection, the mean accumulation effectively smoothed dynamic speckle variations and preserved microvascular continuity without the noise-dilution penalties typically associated with nonmaximum projection methods.^31^

### 2.2 Calculation of Clinical Indicators of OCTA based on Local Fractal Dimension

#### 2.2.1 Local fractal dimension calculation

The fractal dimension (FD) is a robust mathematical metric used to measure the structural complexity of objects. In this study, the box-counting method was adopted due to its proven effectiveness in determining the self-similarity of complex vascular structures across varying dimensions. The fractal dimension $D_{F}$ is defined by the following relationship:^32^

$$D_{F}=\underset{s\to 0}{\lim}\frac{\ln\;N_{s}}{\ln(\frac{1}{s})},$$ (6)

where $N_{s}$ is the number of boxes of size s required to cover the structure in the image. Based on this, the local fractal dimension (LFD) is established in the image by using a sliding window, and the fractal dimension of each pixel in the window is calculated using Eq. (6). Currently, the local fractal dimension has been used to study image texture and surface roughness. The local fractal dimension $D_{\mathrm{LF}}(i,j)$ corresponding to each pixel of the input image I(i,j) in the (2w+1)×(2w+1) window can be calculated using the following method:^32^^–^^34^

$$D_{\mathrm{LF}}(i,j)=\mathrm{FD}[I(i+k,j+k);-w<k<w],$$ (7)

where FD[⋅] represents the fractal dimension calculation method in Eq. (6). To better validate the performance of the fractal dimension method for subsequent clinical metrics, the window size w was carefully selected. If w is too small, the number of pixels containing vessel information is insufficient; conversely, if w is too large, fine capillaries are prone to being overlooked, leading to inaccurate fractal dimension calculations. Therefore, considering the minimum capillary resolution of the current system, the value of w was determined to range from 2 to 6. Specifically, for each localized pixel, the algorithm iterates through this range of kernel sizes, counts the corresponding vessel pixels (white pixels), and performs a logarithmic linear regression. The local fractal dimension is subsequently extracted directly as the slope of this fitted regression line.

#### 2.2.2 Perimeter, area, and circularity index of the foveal avascular zone

The vascular structure of the foveal avascular zone (FAZ) is of great significance for elucidating the pathogenesis of glaucoma. The degeneration or atrophy of capillaries at the macula may affect the shape and size of the FAZ, which has been proven to have diagnostic and prognostic value for retinal diseases such as retinal vein occlusion (RVO) and diabetic retinopathy (DR). The circularity index (CI) of the FAZ is an important indicator for measuring the degree of deviation from a circular shape of the FAZ. It can be calculated based on the perimeter and area of the FAZ shape. The specific calculation formula for the FAZ circularity index $I_{\mathrm{CI}\text{-}\mathrm{FAZ}}$ is as follows:

$$I_{\mathrm{CI}\text{-}\mathrm{FAZ}}=\frac{4\pi \times S_{\mathrm{FAZ}}}{P_{\mathrm{FAZ}}},$$ (8)

where $S_{\mathrm{FAZ}}$ represents the area of the FAZ and $P_{\mathrm{FAZ}}$ represents the perimeter of the FAZ. The value of $I_{\mathrm{CI}\text{-}\mathrm{FAZ}}$ ranges from 0 to 1. The closer the value of $I_{\mathrm{CI}\text{-}\mathrm{FAZ}}$ is to 1, the more circular the shape of the FAZ is; the closer the value of $I_{\mathrm{CI}\text{-}\mathrm{FAZ}}$ is to 0, the less regular the shape of the FAZ is. In addition, the FAZ circularity index and perimeter are among the best discriminant parameters for the eyes of glaucoma patients and healthy controls:^35^

#### 2.2.3 Vessel density

Vessel density index (VD) is an important indicator for assessing retinal vascular abnormalities. Conditions such as diabetic retinopathy (DR), retinal vein occlusion (RVO), sickle cell retinopathy (SCR), age-related macular degeneration (AMD), and glaucoma can all cause abnormal retinal vessels, potentially leading to vascular ischemia and detachment. As the early stages of most diseases are manifested at the capillary level, evaluating retinal vessel density is of great significance.^1^ In this article, VD is defined as the ratio of the vessel area to the area of the region of interest (ROI) in the OCTA image, which can be specifically expressed as:

$$I_{\mathrm{VD}}=\frac{\underset{i,j}{\sum }V(i,j)}{\underset{i,j}{\sum }R(i,j)}\times 100\%,$$ (9)

where V(i,j) represents the area of blood vessels within the ROI and R(i,j) represents the area of the ROI. The higher the value of the vessel’s density $I_{\mathrm{VD}}$, the greater the vessel’s density in that area, and conversely, the lower the density.

#### 2.2.4 OCTA clinical index calculation process

**Fig. 4:**
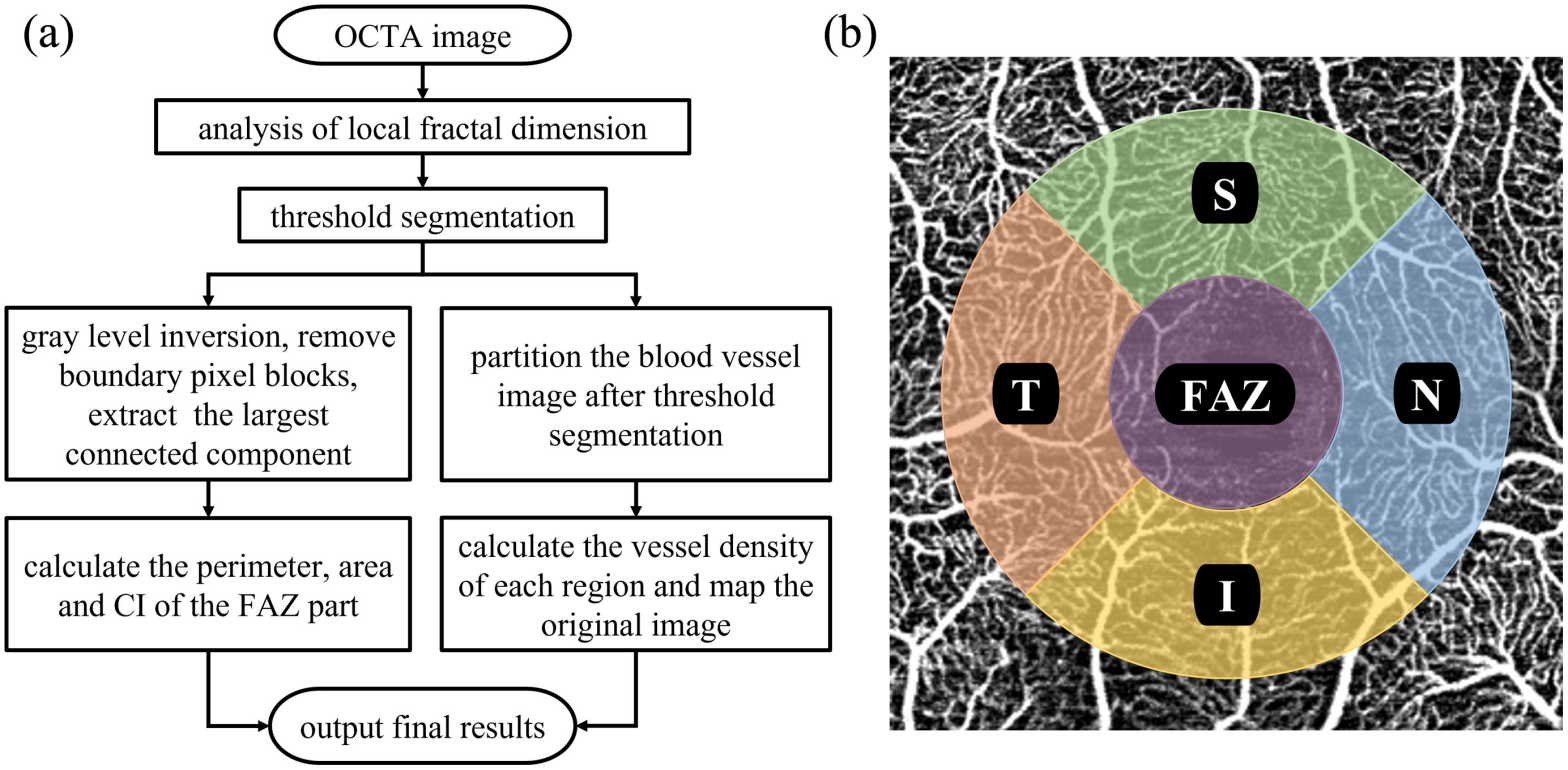
OCTA clinical indicators generation. (a) Flow chart of OCTA clinical index calculation. (b) Schematic diagram of OCTA vessels density zoning.

After obtaining the OCTA images through the OCTA reconstruction algorithm described in the previous chapters, a local fractal dimension analysis was performed on the OCTA images to extract the vessel parts and perform threshold segmentation through adaptive binarization to remove noise.^36^ Subsequently, parallel analysis was conducted on the obtained vessel images. One part was used to extract the FAZ to calculate its perimeter, area, and circularity index. During the extraction of the FAZ, the algorithm precisely applied a sequence of morphological operations. First, a morphological opening operation was utilized to remove isolated noise pixels and fine boundary artifacts from the binarized mask. Following a gray-scale inversion to highlight the avascular spaces, the algorithm automatically extracted the largest connected component, which reliably corresponds to the FAZ region. These precise steps allowed for the automated calculation of its perimeter, area, and circularity index without manual intervention. For the other part, the OCTA image after extracting the vessels was partitioned into each region, and the vessel density was calculated separately in each region. In this paper, according to the method described in Ref. 33, the circular region centered on the FAZ was first excluded, and the peripheral field around this circular region was divided into four regions: temporal (temporal, T), superior (superior, S), nasal (nasal, N), and inferior (inferior, I). Figure 4(a) shows the flowchart for calculating the OCTA clinical indicators, and Fig. 4(b) shows the OCTA image of the retinal IR vessels plexus and the schematic diagrams of the four peripheral field regions (T, S, N, I).

#### 2.3 Evaluation and Quantification Metrics

To quantitatively evaluate the performance of the proposed reconstruction algorithm and the accuracy of the automated clinical indicator extraction, several standard metrics were employed. First, for image quality assessment, the analysis indicators included vessel connectivity, contrast, and signal-to-noise ratio (SNR).^37^ According to the robust evaluation methodology established in Ref. 36, the OCTA images before and after enhancement were strictly averaged to generate a fused vessel mask image M(i,j), which was subsequently skeletonized to a 1-pixel width. This averaged mask image served as the “ground truth.” It is important to emphasize that employing manual annotation for image quality assessment presents significant challenges; particularly in low-SNR OCTA images, microvessels are often indistinguishable from background noise, leading to severe subjective variability. By utilizing an averaging approach, we effectively eliminate the bias introduced by subjective human judgment. Furthermore, it ensures that the ground truth image maintains a balanced, equidistant baseline from all evaluated OCTA reconstructions, providing a fair and objective standard for calculating metrics such as contrast and SNR. The specific formula for calculating vessel connectivity $V_{\text{connectivity}}$ is as follows:

$$V_{\text{connectivity}}=\mathrm{Std}[I(i,j){|}_{M(i,j)=1}],$$ (10)

where Std[⋅] is the standard deviation calculation function; I(i,j) is the OCTA image. The vessel connectivity defined by this equation is expressed as the standard deviation of the intensity of the OCTA image that completely overlaps with the mask image. The lower the value, the better the vessel connectivity in the OCTA image. The contrast $V_{\text{contrast}}$ of the OCTA image is calculated as follows:

$$V_{\text{contrast}}=\frac{\text{Mean}[I(i,j){|}_{M(i,j)=1}]}{\mathrm{Std}[I(i,j){|}_{M(i,j)=1}]},$$ (11)

where Mean[⋅] is the mean calculation function. The OCTA image contrast defined by this formula is expressed as the ratio of the mean intensity of the OCTA image that completely overlaps with the mask image to the standard deviation. The higher the value, the higher the OCTA image contrast. The formula for calculating the signal-to-noise ratio $V_{\mathrm{SNR}}$ of the OCTA image is as follows:

$$V_{\mathrm{SNR}}=10\;\mathrm{lg}\{\frac{\text{Mean}[I(i,j){|}_{M(i,j)=1}]}{\mathrm{Std}[I(i,j){|}_{N(i,j)=1}]}\},$$ (12)

where N(i,j) is the background mask image obtained by dilating and reversing M(i,j) using a 3×3 circular structuring element. The signal-to-noise ratio of the OCTA image defined by this formula is the ratio of the mean intensity of the OCTA image that completely overlaps with the mask image to the standard deviation of the OCTA image that completely overlaps with the background mask image. Here, the width of the vessels in the background image is comparable to the width of the large vessels in the OCTA image. The higher the value of $V_{\mathrm{SNR}}$, the higher the signal-to-noise ratio of the OCTA image.

Furthermore, recognizing that intensity-based connectivity metrics may be partially confounded by physiological variations in blood flow when comparing different algorithms, we introduced a purely morphological metric to evaluate structural connectivity directly: the largest connected component (LCC) ratio. To compute the LCC ratio, the OCTA image was first binarized using adaptive local thresholding and then processed with a morphological thinning algorithm to extract a skeletonized vascular network (1-pixel width). The LCC ratio is defined as the total number of pixels within the single largest interconnected vascular tree divided by the total number of vascular skeleton pixels in the entire image. A higher LCC ratio strictly indicates fewer fragmented capillaries and superior structural continuity, effectively isolating structural integrity from blood flow intensity.

Second, for the evaluation of clinical indicators, specifically to verify the accuracy of FAZ segmentation, 20 OCTA images of 10 volunteers were manually annotated by professional image readers to form the ground truth for the FAZ area. Because the FAZ boundary in the foveal region is morphologically distinct, manual annotation by experts provides a highly reliable and accurate reference for evaluating this specific macroscopic clinical indicator. Then, the similarity index and segmentation error between the algorithm in this paper and the ground truth results were analyzed to verify the accuracy of the local fractal dimension for segmenting the FAZ area. The similarity index is also called the kappa coefficient, which measures the similarity between two segmented regions by the ratio of the intersection area to the combined area of the two regions. The similarity index $k_{\mathrm{FAZ}}$ for the FAZ area can be expressed as

$$k_{\mathrm{FAZ}}=\frac{2|S_{\mathrm{GT}}\cap S_{\mathrm{LFD}}|}{\left|S_{\mathrm{GT}}|+|S_{\mathrm{LFD}}\right|},$$ (13)

where $S_{\mathrm{GT}}$ represents the area of the FAZ region manually annotated by OCTA, and $S_{\mathrm{LFD}}$ represents the area of the FAZ region segmented based on the local dimension algorithm. The value of $k_{\mathrm{FAZ}}$ lies between 0 and 1; 0 indicates that the two regions do not have overlapping parts, whereas 1 indicates that the two regions are completely overlapping. In addition, this paper determines the segmentation error by calculating the false positive rate $r_{P}$ and the false negative rate $r_{N}$. Here, the false positive rate represents the probability of non-FAZ regions being included in the automatic segmentation result, and vice versa. The calculation methods for the false positive rate and the false negative rate are to divide the area of the incorrectly segmented non-FAZ regions and the FAZ regions by the area of the FAZ region in the ground truth. The false positive rate $r_{P}$ and the false negative rate $r_{N}$ are respectively as shown in Eqs. (14) and (15).^38^

$$r_{P}=\frac{\left|S_{\mathrm{LFD}}|-|S_{\mathrm{GT}}\cap S_{\mathrm{LFD}}\right|}{\left|S_{\mathrm{GT}}\right|}\times 100\%,$$ (14)

$$r_{N}=\frac{\left|S_{\mathrm{GT}}|-|S_{\mathrm{GT}}\cap S_{\mathrm{LFD}}\right|}{\left|S_{\mathrm{GT}}\right|}\times 100\%,$$ (15)

To this end, the intraclass correlation coefficient (ICC) among the perimeter, area, and circularity index of the segmented regions by the automatic segmentation algorithm and the ground truth was calculated. The values range from 0 to 1, with 0 indicating complete unreliability and 1 indicating complete reliability.^39^

## 3 OCTA Reconstruction and Clinical Index Calculation Experiment

### 3.1 Setup of the OCT System

The OCTA data in this study were collected using a custom-built spectral-domain optical coherence tomography (SD-OCT) system. The system utilizes a superluminescent diode (SLD, Suzhou Bofu Photoelectric Technology Co., Ltd., Jiangsu, China) as the low-coherence light source, with a central wavelength of 840  nm±10  nm and a half-wavelength bandwidth greater than 40 nm. The backscattered light from the sample and the reference mirror is detected by a high-speed spectrometer camera (LUSTER LightTech Co., Ltd., Beijing, China) to generate high-resolution tomographic images. This study was approved by the Institutional Review Board (IRB) and obtained approval from the Ethics Review Committee of Sichuan Provincial People’s Hospital. All volunteers provided informed consent through a process reviewed and approved by the IRB.

### 3.2 Results of the OCTA Reconstruction Experiment

#### 3.2.1 Validation of the Walsh function smoothing algorithm

**Fig. 5:**
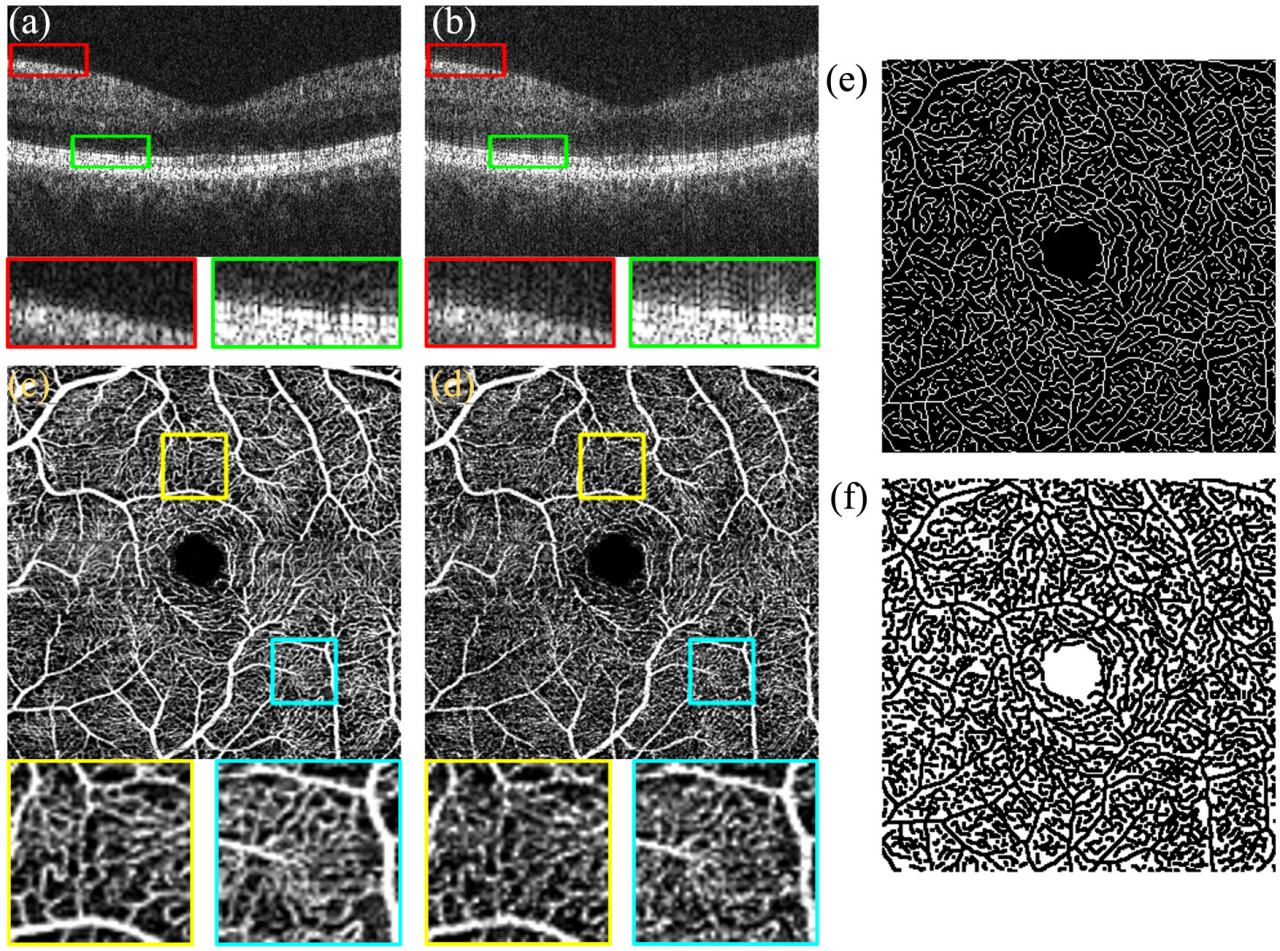
Comparison of OCTA reconstruction results before and after Walsh function smoothing. (a) Amplitude image with smoothed fourth Walsh function post-FFT. (b) Amplitude image with unsmoothed fourth Walsh function post-FFT. (c) OCTA image of the IR layer reconstructed using the smoothed Walsh function. (d) OCTA image of the IR layer reconstructed using the unsmoothed Walsh function. (e) “Ground truth” of the vessel network in the IR layer of the OCTA image. (f) “Ground truth” of the background in the IR layer of the OCTA image.

To investigate the differences between the proposed method and the original Walsh function, we partitioned the raw spectrum using both the unsmoothed and smoothed Walsh window functions, respectively, and subsequently reconstructed the OCTA images using the method proposed in this study. Based on the evaluation metrics defined in Sec. 2.3, the OCTA images of the Inner Retina (IR) layer were analyzed, and the results are presented in Table 1. This table summarizes the mean ± standard deviation (SD) of image quality metrics derived from 20 retinal volumes obtained from 10 volunteers, comparing reconstructions using the smoothed versus unsmoothed Walsh functions. The OCTA images reconstructed with the smoothed function exhibited superior vessel connectivity, contrast, and SNR compared with the unsmoothed versions. Specifically, the contrast improved by ∼0.15, and the SNR increased by ∼1.48  dB. Furthermore, as illustrated in Fig. 5, the OCTA images reconstructed using the smoothed Walsh function demonstrate superior visual quality. Figure 5(a) shows the amplitude image for blood flow extraction obtained after windowing with the smoothed 4th Walsh window function and performing FFT, whereas Fig. 5(b) displays the corresponding amplitude image obtained using the 4th Walsh window function without the smoothing operation. Figure 5(c) presents the OCTA image reconstructed with the smoothed Walsh function, and Fig. 5(d) presents the OCTA image reconstructed without smoothing. Figure 5(e) depicts the “ground truth” of the vessel network in the IR layer generated using the proposed method, and Fig. 5(f) depicts the “ground truth” of the nonvessel background region in the IR layer.

**Table 1:** Mean ± standard deviation of quality assessment metrics for OCTA images of the IR layer before and after Walsh function smoothing.

Walsh function smooth	Connectivity $V_{\text{Connectivity}}$		Contrast $V_{\text{Contrast}}$		SNR $V_{\mathrm{SNR}}$ /dB	
	Smooth	Unsmooth	Smooth	Unsmooth	Smooth	Unsmooth
IR	62.36 ± 1.59	66.57 ± 1.09	2.49 ± 0.09	2.34 ± 0.04	5.39 ± 0.19	3.91 ± 0.17

#### 3.2.2 Validation of the OCTA blood flow B-scan enhancement algorithm

During the acquisition of OCTA data, if the patient’s eye movement or other internal factors of the acquisition machine cause changes in the axial position of the OCT reflection structure or blood flow, it may lead to a decrease in the correlation of the dynamic signals, resulting in them being wrongly detected as static signals. Conversely, static signals may also be detected as dynamic signals. The extracted OCTA blood flow B-scan signals were analyzed using the OCTA blood flow B-scan enhancement algorithm described in the previous section. The analysis metrics included vessel connectivity, contrast, and SNR.

**Fig. 6:**
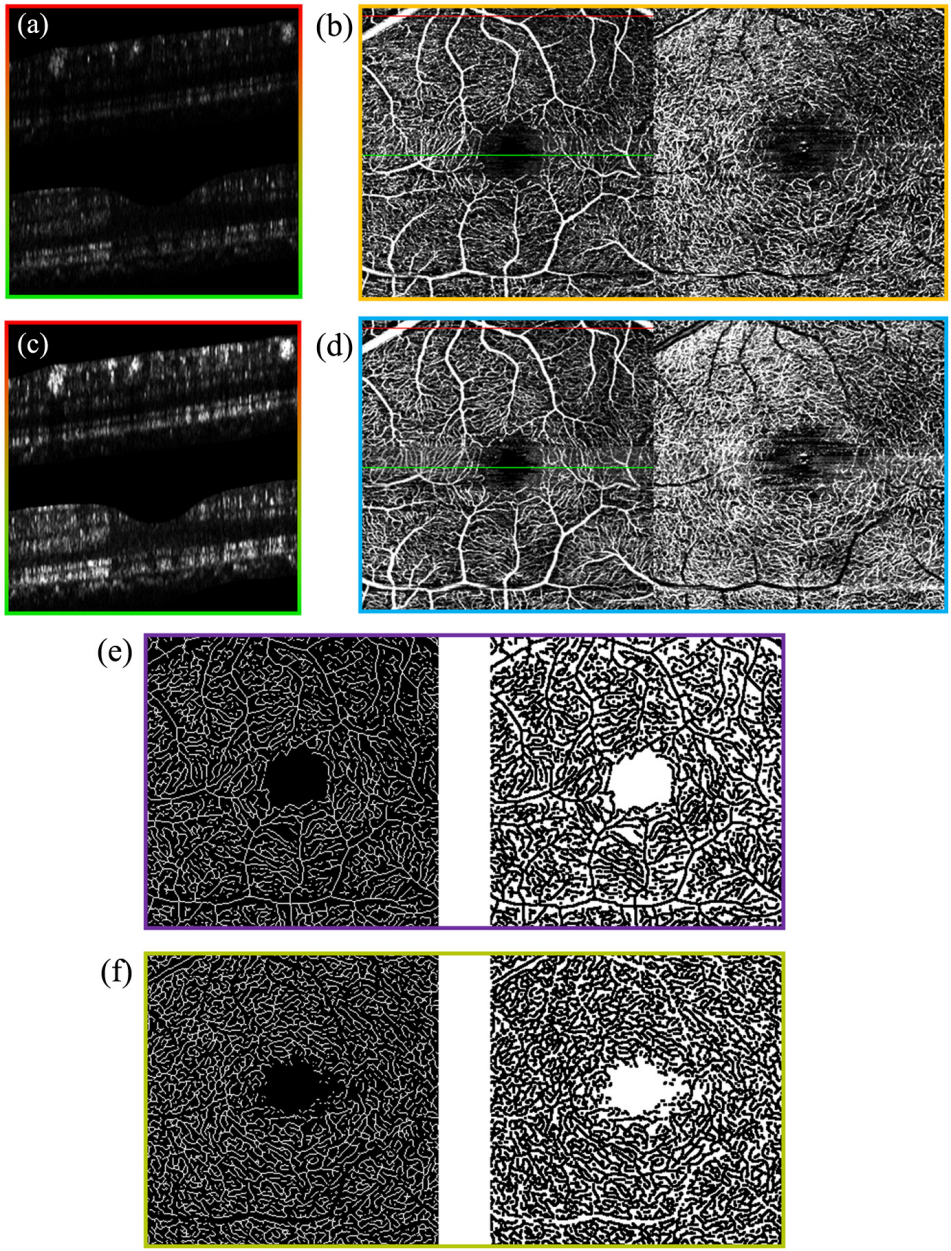
Effect of OCTA reconstruction before and after blood flow B-scan image enhancement. (a) Unenhanced blood flow B-scan images. (b) OCT images reconstructed by unenhanced blood flow signals. (c) Enhanced blood flow B-scan images. (d) OCT images of enhanced blood flow signal reconstruction. (e) The “ground truth” of blood vessels and background in the OCTA image of the IR layer. (f) The “ground truth” of blood vessels and background in the OCTA image of the IR layer.

Using the methodology described in the previous section, the OCTA images of the IR and MR layers were analyzed, respectively. The results are presented in Table 2. This table summarizes the mean ± standard deviation of image quality metrics derived from 20 retinal volumes obtained from 10 volunteers, specifically comparing reconstructions with and without the B-scan blood flow enhancement step. Across both the IR and MR layers, the enhanced OCTA images exhibited superior vessel connectivity, contrast, and SNR compared with the unenhanced versions. Specifically, the IR layer demonstrated an improvement of ∼0.37 in contrast and 1.20 dB in SNR, whereas the MR layer showed an increase of ∼0.52 in contrast and 0.75 dB in SNR. In addition, as can be seen from Fig. 6, the effect of using the method described in this paper to enhance the blood flow B-scan and obtain the OCTA image is better. Figure 6(a) shows the unenhanced blood flow B-scan image, and the red-green gradient from top to bottom corresponds to the blood flow B-scan images of the red line and green line in Fig. 6(b); Fig. 6(b) shows the OCTA projection obtained by reconstructing the unenhanced blood flow signal, with the left side being the OCTA image of the IR layer and the right side being the OCTA image of the MR layer; Fig. 6(c) shows the enhanced blood flow B-scan image, with the red-green gradient from top to bottom corresponding to the blood flow B-scan images of the red line and green line in Fig. 6(d); Fig. 6(d) shows the OCTA projection obtained by reconstructing the enhanced blood flow signal, with the left side being the OCTA image of the IR layer and the right side being the OCTA image of the MR layer; Fig. 6(e) displays, from left to right, the “ground truth” of the vessel network and the “ground truth” of the nonvessel background region in the IR layer, generated using the method described in the previous section. Figure 6(f) displays, from left to right, the “ground truth” of the vessel network and the “ground truth” of the nonvessel background region in the MR layer, generated using the same method.

**Table 2:** Mean ± standard deviation of OCTA image quality metrics before and after B-scan blood flow signal enhancement.

Blood flow enhance	Connectivity $V_{\text{Connectivity}}$		Contrast $V_{\text{Contrast}}$		SNR $V_{\mathrm{SNR}}$ /dB	
	Original	Enhanced	Original	Enhanced	Original	Enhanced
IR	67.90 ± 2.17	62.36 ± 1.59	2.12 ± 0.13	2.49 ± 0.09	4.19 ± 0.16	5.39 ± 0.19
MR	64.53 ± 1.42	57.83 ± 1.66	2.44 ± 0.08	2.96 ± 0.13	4.35 ± 0.23	5.10 ± 0.15

#### 3.2.3 Comparison of performance of different OCTA reconstruction algorithms

**Fig. 7:**
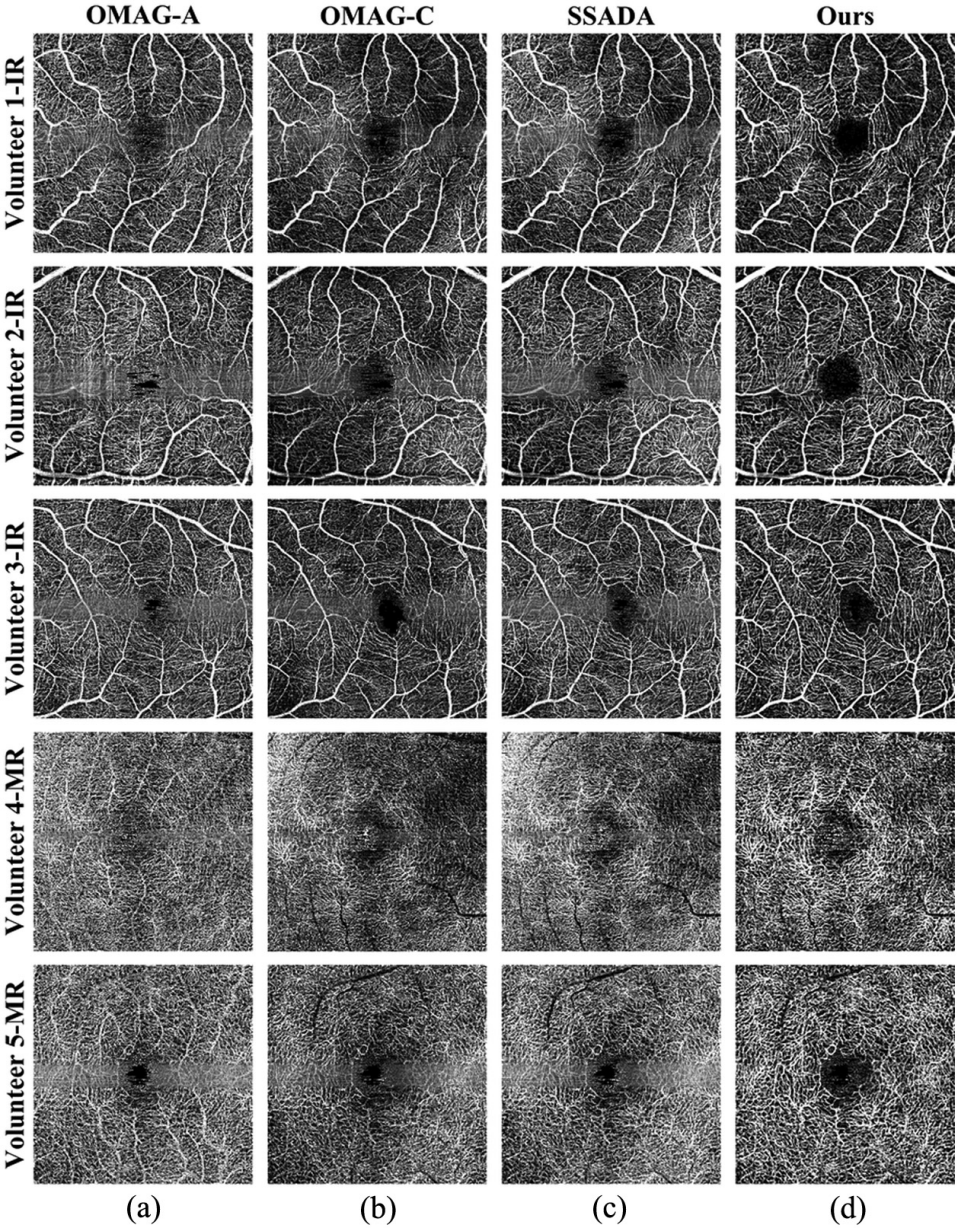
Comparison of OCTA images reconstructed by the proposed algorithm and other algorithms. (a) Reconstruction results of the OMAG algorithm based on intensity. (b) Reconstruction results of the OMAG algorithm based on complex numbers. (c) SSADA reconstruction results. (d) Reconstruction results of this algorithm.

To demonstrate the superiority of the performance of the algorithm in this paper, the OCTA images reconstructed by the OMAG-A algorithm, the OMAG-C algorithm, and the SSADA algorithm were compared with those of the algorithm in this paper. All algorithms adopted the same original spectral signal processing method, OCT layering algorithm, and projection operations. In addition, for the SSADA algorithm, we utilized 11 Gaussian windows to partition the raw spectrum, with each Gaussian window having a full width at half maximum (FWHM) of 15 nm. The OCTA image quality evaluation standard described in the previous section was still used to analyze all the OCTA images processed by the four different algorithms. The “ground truth” image was generated by averaging the four OCTA images, and the weights of the four OCTA images were consistent. The analysis results are shown in Table 3, which presents the mean ± standard deviation of the quality assessment indicators of different layers of OCTA images reconstructed by the different algorithms for the 20 retinal data of 10 volunteers. It can be seen from the table that the algorithm proposed in this paper outperforms the other three algorithms in terms of vessel connectivity, contrast, and SNR. Furthermore, the morphological evaluation based on the LCC Ratio demonstrates that our method achieves superior structural connectivity in both the IR and MR layers. This morphological metric confirms that the observed image quality enhancements reflect a highly continuous physical vessel reconstruction, rather than being confounded by variable blood flow dynamics or background noise. Figure 7 shows the OCTA images obtained from the different layers of the retina of some volunteers using different OCTA reconstruction algorithms. It can be seen from the figure that the algorithm proposed in this paper reconstructs the capillary connectivity better and is clearer, and the visual quality of the image is the best. Furthermore, a noticeable horizontal blurring stripe is apparent across the central foveal region in the images reconstructed by the OMAG and SSADA algorithms. This artifact arises because blood flow signals in B-scans at the foveal avascular center are inherently weak; when conventional uniform enhancement is applied, background noise in this avascular zone is inevitably amplified, manifesting as a bright, blurred band upon en face projection. By contrast, our proposed method integrates precise layer segmentation to effectively mask out nonvascular noise and strictly suppress this over-enhancement. Consequently, the reconstructed OCTA images generated by our approach maintain a uniformly dark avascular background, successfully eliminating this artifact and further demonstrating the superior visual quality of our algorithm.

**Table 3:** Mean ± standard deviation of OCTA image quality evaluation indexes of different layers reconstructed by different algorithms.

Algorithm	Connectivity $V_{\text{Connectivity}}$		LCC Ratio (%)		Contrast $V_{\text{Contrast}}$		SNR $V_{\mathrm{SNR}}$ (dB)	
	IR	MR	IR	MR	IR	MR	IR	MR
OMAG-A	64.93 ± 3.04	64.93 ± 2.96	88.06 ± 4.05	86.69 ± 3.61	2.29 ± 0.26	2.36 ± 0.27	4.58 ± 0.10	4.01 ± 0.19
OMAG-C	67.74 ± 1.94	66.59 ± 2.38	80.48 ± 5.56	79.00 ± 9.95	2.03 ± 0.17	2.22 ± 0.18	4.63 ± 0.21	4.09 ± 0.22
SSADA	65.13 ± 1.40	63.84 ± 1.88	89.01 ± 3.60	87.87 ± 7.13	2.30 ± 0.07	2.48 ± 0.14	4.72 ± 0.14	4.21 ± 0.27
Ours	62.36 ± 1.59	57.83 ± 1.66	90.32 ± 4.69	88.89 ± 3.80	2.49 ± 0.09	2.96 ± 0.13	5.39 ± 0.19	5.10 ± 0.15

To further validate the statistical superiority of the proposed method, we performed paired sample t-tests and effect size analyses on the image quality metrics of OCTA images reconstructed by different algorithms. Table 4 presents the results of the significance tests, demonstrating that the proposed method exhibits statistically significant differences compared with the other three algorithms (OMAG-A, OMAG-C, and SSADA) across all three metrics: vessel connectivity, contrast, and signal-to-noise ratio (SNR) (mostly P<0.05, with some P<0.001). Furthermore, to quantify the practical magnitude of these performance improvements, we calculated Cohen’s d as an indicator of effect size (Table 5). The results reveal that the Cohen’s d values for the comparisons between the proposed method and the other algorithms are consistently greater than 0.8 (ranging from 0.979 to 6.649). This indicates that the proposed reconstruction method not only is statistically superior to existing methods but also demonstrates a large effect size in terms of image quality improvement, thereby significantly enhancing the interpretability of clinical images.

**Table 4:** Paired sample t-test results (P-values) of OCTA image quality metrics reconstructed by different algorithms.

P-value (paired)	Connectivity		Contrast		SNR	
	IR	MR	IR	MR	IR	MR
OMAG-A versus ours	0.028	<0.001	0.038	<0.001	<0.001	<0.001
OMAG-C versus ours	0.001	0.018	0.018	0.018	<0.001	<0.001
SSADA versus ours	<0.001	0.018	<0.001	<0.001	<0.001	<0.001

**Table 5:** Effect size analysis (Cohen’s d) of the proposed method compared with other algorithms.

Cohen’s d	Connectivity		Contrast		SNR	
	IR	MR	IR	MR	IR	MR
OMAG-A versus ours	0.979	3.137	0.945	2.666	6.649	5.825
OMAG-C versus ours	2.215	3.943	3.085	4.179	3.440	4.843
SSADA versus ours	3.445	3.132	6.017	6.473	3.631	6.568

### 3.3 OCTA Clinical Index Calculation Result Experiment

#### 3.3.1 Verification of the calculation results for the perimeter, area, and circularity index of FAZ

**Fig. 8:**
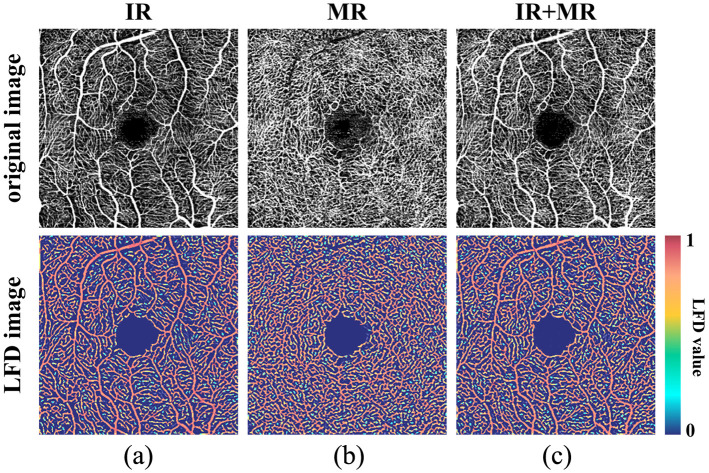
Visualization results of the local fractal dimension of the OCTA image. (a) Visualization results of local fractal dimension in the IR layer. (b) Visualization results of the local fractal dimension of the MR layer. (c) Visualization results of the local fractal dimension of the IR + MR mixing layer.

To calculate the relevant indicators of FAZ, the first step was to segment the OCTA images to extract the morphological features of the contour vessels at the FAZ. After performing local dimensionality calculations on the OCTA images, the potential noise signals were removed using the threshold segmentation method described in the previous section. Then, the local fractal dimensions were normalized for further analysis. Figure 8 shows the local fractal dimension graphs of the IR layer, MR layer, and IR + MR mixed layer of a set of OCTA images. From the figure, it can be observed that the local shape dimensions of small vessels and capillaries are close to 1, whereas those of nonvessel areas are close to 0. Larger vessels have the highest local shape dimensions.

**Fig. 9:**
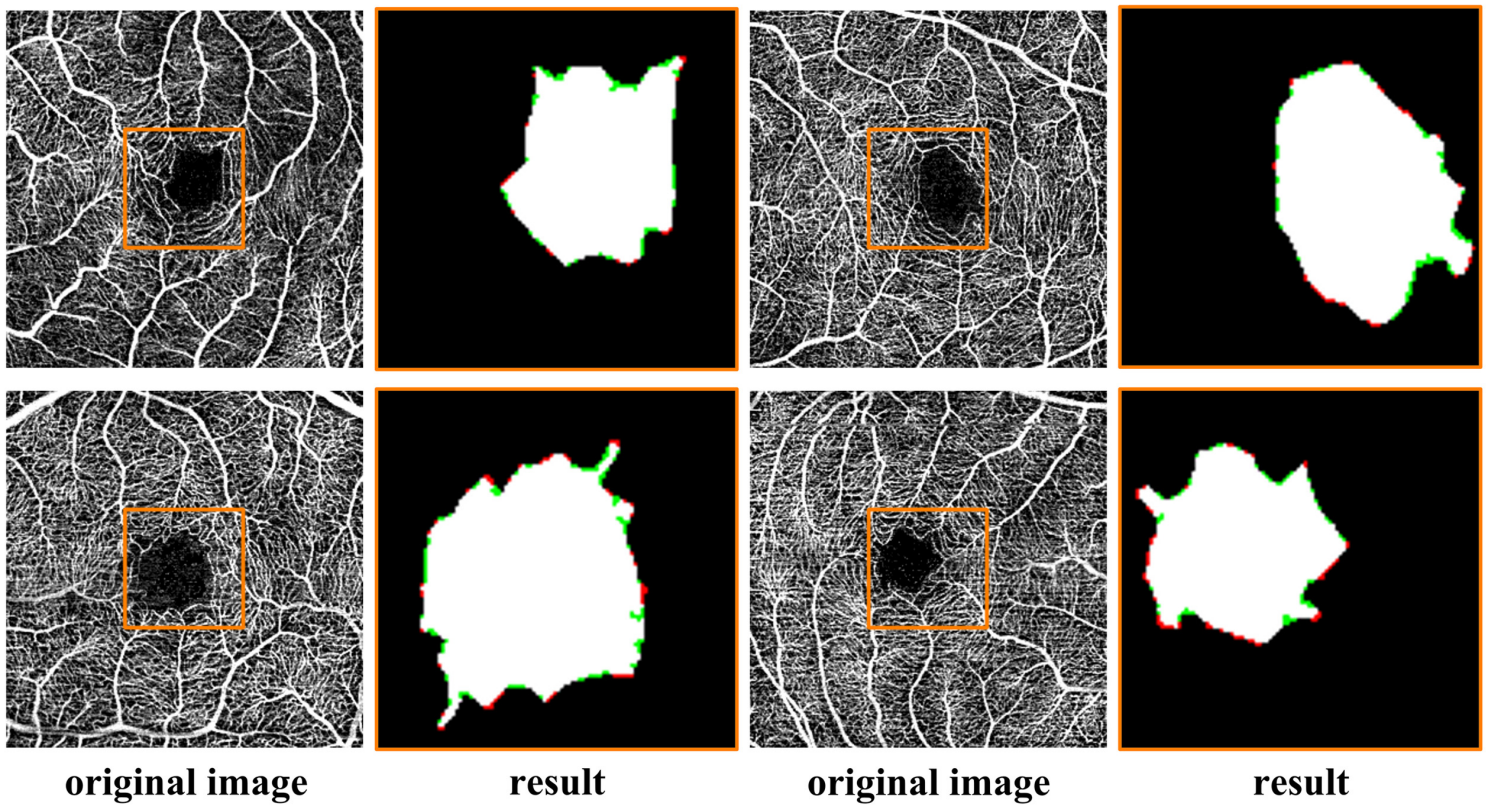
Visual results of automatic segmentation of the FAZ region and comparison with ground truth.

To better segment the FAZ area and reduce the impact of layering on the vessel connectivity around the FAZ, this paper used the local fractal dimension results of the IR + MR mixed-layer OCTA images to extract the vessel structures near the FAZ. Figure 9 shows the results of automatic segmentation of the FAZ area in four representative OCTA images. In the segmentation images, the white part represents the overlapping area between the automatically segmented region and the ground truth region, the green part represents the area that the ground truth classifies as an FAZ region but the automatic segmentation algorithm fails to segment, and the red part indicates the area that the ground truth classifies as non-FAZ but is segmented by the automatic segmentation algorithm.

Based on the metrics defined in Sec. 2.3, Table 6 shows the average similarity index and segmentation error between the ground truth and the automatic segmentation of the FAZ in 20 OCTA images. From the table, it can be seen that the automatic segmentation algorithm based on local fractal dimension for the FAZ performs well in segmenting the FAZ area. As shown in Table 6, the similarity index of its segmentation results compared with the ground truth is 0.9735±0.0066, the false positive rate is 1.7621%±0.7465%, and the false negative rate is 3.4834%±0.7784%.

**Table 6:** Average similarity index and segmentation error between ground truth and automatic segmentation of the FAZ region.

Segmentations	Similarity index $k_{\mathrm{FAZ}}$	False-positive rate $r_{P}$ (%)	False-negative rate $r_{N}$ (%)
FAZ	0.9735±0.0066	1.7621±0.7465	3.4834±0.7784

Using the results of the aforementioned automatic segmentation to calculate the relevant clinical indicators of FAZ, this study aims to determine the applicability of the automatic segmentation algorithm’s results for calculating the FAZ indicators. Table 7 shows the ICC used to evaluate the degree of agreement between the manually segmented FAZ regions and the FAZ regions calculated using the automated segmentation algorithm. From the table, it can be seen that the clinical indicators calculated for the FAZ regions segmented by the automated algorithm have a high correlation with the ground truth and can be fully used as a method for FAZ region segmentation. In addition, the area distribution of the FAZ regions in all ground truths in this study was $0.3392\;{\mathrm{mm}}^{2}\pm 0.0921\;{\mathrm{mm}}^{2}$, whereas the area distribution of the FAZ regions segmented by the automated algorithm was $0.3301\;{\mathrm{mm}}^{2}\pm 0.0906\;{\mathrm{mm}}^{2}$. This result is comparable to some previous research findings. In a study report on the OCTA FAZ regions of healthy subjects, it was pointed out that the average superficial FAZ region area was $0.358\;{\mathrm{mm}}^{2}\pm 0.084\;{\mathrm{mm}}^{2}$, and the average deep FAZ region area was $0.584\;{\mathrm{mm}}^{2}\pm 0.15\;{\mathrm{mm}}^{2}$.^34^

**Table 7:** Intra group correlation coefficient of FAZ segmentation by different methods.

Intraclass correlation coefficient	Area of FAZ $S_{\mathrm{FAZ}}$ (${\mathrm{mm}}^{2}$)	Perimeter of FAZ $P_{\mathrm{FAZ}}$ (mm)	Circularity Index of FAZ $I_{\mathrm{CI}\text{-}\mathrm{FAZ}}$
ICC	0.9951	0.9727	0.9022

#### 3.3.2 Verification of OCTA vessel density calculation results

**Fig. 10:**
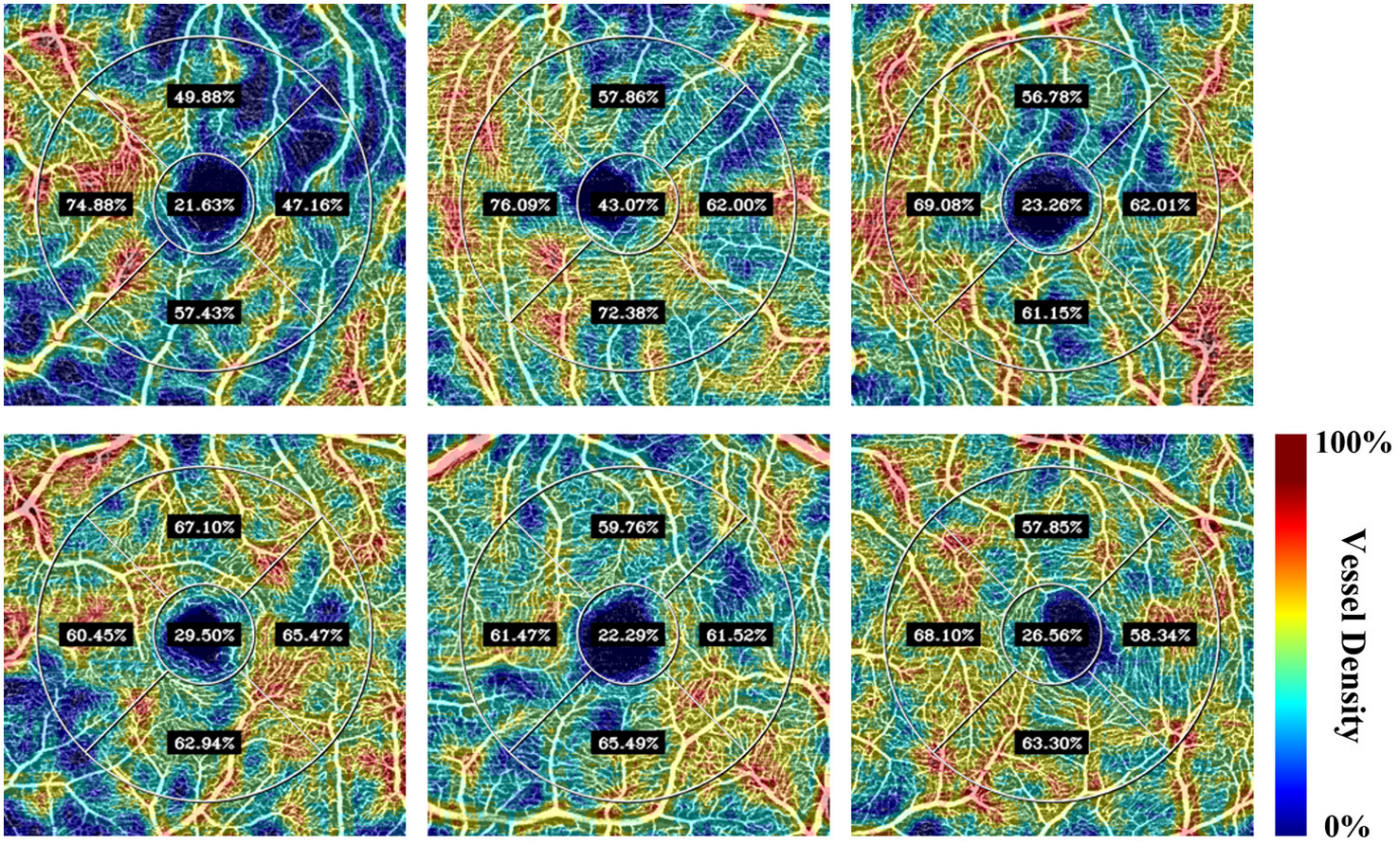
Color-coded mapping of vessel density.

The previous section demonstrated the powerful performance of the local fractal dimension-based automated algorithm in extracting the FAZ region. This section continued to use the vessels segmentation based on the local fractal dimension algorithm to calculate the vessels density of each partition as described in the previous sections. In addition, this section only analyzed the vessel density of the IR layer. Table 8 shows the average vessel density of each partition of the IR layer in 10 volunteers’ 20 eyes. It should be noted that for the verification of the vessel density (VD) analysis, a traditional pixel-level manual annotation was not utilized, as manually tracing dense capillary networks across a large field of view is practically infeasible and highly prone to human error. Instead, our automated quantification was verified against macroscopic physiological norms established in prior clinical literature. From the table, it can be seen that the vessel density in zone I is the highest. This regional density distribution is highly consistent with the normative research results reported by Gadde et al.,^34^ thereby demonstrating the reliability and physiological accuracy of our automated VD calculation pipeline. Figure 10 presents the color mapping of OCTA vessels density for some volunteers, where the transition from blue to red represents the increase in vessel density.

**Table 8:** Mean ± standard deviation of OCTA vessel density in different regions of the IR layer.

Region	Vessel density $I_{\mathrm{VD}}$ (%)
T	67.17 ± 2.37
S	56.26 ± 1.94
N	61.54 ± 1.30
I	71.33 ± 1.71

## 4 Discussion

Our proposed OCTA reconstruction algorithm demonstrated superior performance compared with established methods such as OMAG and SSADA. Specifically, it achieved improved vascular connectivity, contrast, and SNR in both the IR and MR layers. The maximum gains were ∼0.74 in contrast and 1.09 dB in SNR. These enhancements are attributed to the use of a smoothed Walsh window, which reduces spectral leakage while preserving axial resolution, and the integration of a retinal layer segmentation algorithm that optimizes the threshold for blood flow signal enhancement. Crucially, the success of this fully automated, and LFD-based clinical quantification pipeline is intrinsically dependent on the high-fidelity OCTA images generated in the first part of this study. The proposed smoothed Walsh reconstruction method significantly improves microvascular continuity and contrast while suppressing noise artifacts. This superior structural integrity serves as the fundamental prerequisite, ensuring that the subsequent LFD analysis accurately delineates the FAZ boundary and avoids the segmentation errors that typically arise from capillary dropouts or spectral leakage in standard reconstruction algorithms. In the automated analysis of clinical indicators, the local fractal dimension-based approach achieved a high similarity index (0.9735) with manual FAZ annotations, along with low false positive (1.76%) and false negative (3.48%) rates. The ICC values for FAZ area, perimeter, and circularity index all exceeded 0.90, indicating excellent agreement with ground truth. Furthermore, vessel density measurements across different retinal sectors were consistent with previously reported values in healthy subjects.^34^

The improved Walsh window function effectively mitigates the jump discontinuities inherent in the original Walsh function, thereby reducing spectral leakage—a known source of artifact in OCTA imaging.^28^ This adjustment allows for more accurate extraction of decorrelation signals without significantly compromising axial resolution. The combination of this window with a deep-learning-based layer segmentation algorithm further refines the blood flow signal by focusing on anatomically relevant regions, leading to clearer visualization of microvasculature.

The high accuracy of FAZ segmentation using local fractal dimension underscores its utility in capturing complex vascular patterns. Fractal dimension has been widely used to characterize biological structures due to its sensitivity to texture and complexity.^32^^,^^34^ Our results confirm that LFD can reliably distinguish vascular from nonvascular regions, facilitating automated and objective FAZ quantification. This is particularly valuable in diseases such as diabetic retinopathy and glaucoma, where FAZ morphology serves as a key biomarker.^35^ Vessel density measurements derived from our method align well with existing literature,^34^ supporting the validity of the proposed partitioning scheme. The automated nature of this process reduces inter-observer variability and enhances reproducibility, which is critical for longitudinal studies and clinical trials.

Several studies have explored OCTA reconstruction and quantification. For instance, Gadde et al.^34^ also employed local fractal dimension for vessel density quantification and reported comparable values in healthy retinas. However, their method did not integrate an enhanced reconstruction process. Similarly, Gao et al.^18^ introduced SSADA but noted its limitations in axial resolution due to spectrum splitting. Our approach addresses this using a full-spectrum Walsh window, thereby maintaining better resolution while improving motion contrast. In terms of FAZ analysis, Choi et al.^35^ highlighted the diagnostic value of FAZ circularity in glaucoma. Our automated method achieves ICC values above 0.90 for all FAZ parameters, which is comparable to or higher than those reported in earlier semi-automated studies.^38^ This suggests that fully automated pipelines can match human performance in certain tasks.

Recently, a deep-learning-based spatial vascular connectivity network (SVC-Net) was proposed to construct OCTA images from single volumetric OCT scans, potentially improving clinical applicability by enhancing lateral resolution or expanding the field of view.^40^ Prior to this, a weakly supervised deep-learning-based OCTA reconstruction method was introduced; employing a cross-validation strategy without the need for high-quality training labels, its performance was validated on *in vivo* animal and human datasets, demonstrating outcomes comparable or even superior to supervised learning methods.^41^ Although these studies indicate that significant progress has been made in deep-learning-based OCTA reconstruction algorithms, to date, no commercial OCT system has incorporated such algorithms. Furthermore, whether deep learning induces the generation of spurious dynamic blood flow signals during reconstruction remains a critical issue that warrants further investigation.

Although our method yielded OCTA results superior to those generated by widely used reconstruction algorithms, this study is subject to certain limitations. Future work will focus on the reconstruction of pathological OCTA volumetric data to further validate the robustness and advanced nature of our proposed method, thereby enhancing its clinical applicability.

## 5 Conclusion

This paper presents an OCTA reconstruction method based on the improved Walsh window function and an automatic calculation method for OCTA clinical indicators based on local fractal dimension and morphology combination. By constructing a smoothed Walsh window function, the extraction accuracy of blood flow signals has been effectively improved; and by combining the retinal layering algorithm to optimize the segmentation threshold, the intensity of blood flow B-scan signals has been enhanced, further improving the quality of OCTA images. The experimental results demonstrate that, following Walsh function smoothing, the contrast of the IR layer improved by a maximum of ∼0.15, and the SNR increased by a maximum of ∼1.48  dB. Furthermore, after blood flow B-scan enhancement, the contrast of the IR and MR layers improved by maximums of ∼0.37 and 0.52, respectively, whereas the SNR increased by maximums of ∼1.20 and 0.75 dB, respectively. At the same time, the OCTA reconstruction algorithm proposed in this paper outperforms the traditional OMAG and SSADA methods in terms of vessel connectivity, contrast, and signal-to-noise ratio. Moreover, compared with the ground truth, the similarity index of the automatic segmentation algorithm based on local fractal dimension FAZ is 0.9735±0.0066, the false positive rate and false negative rate are 1.76%±0.75% and 3.48%±0.78%, respectively, and the intra-group correlation coefficients of the calculated FAZ area, perimeter, and near-circularity index are all higher than 0.90. The vessel density partition results are also comparable to the existing research results. This study provides reliable technical support for the reconstruction of OCTA volumetric data and the automatic analysis and clinical application of OCTA images.

## Data Availability

Data underlying the results presented in this paper are not publicly available at this time but may be obtained from the authors upon reasonable request.
